# Perceived barriers and enablers to participation in a community-tailored physical activity program with Indigenous Australians in a regional and rural setting: a qualitative study

**DOI:** 10.1186/s12939-017-0664-1

**Published:** 2017-09-18

**Authors:** Ashleigh Sushames, Terry Engelberg, Klaus Gebel

**Affiliations:** 1 Centre for Chronic Disease Prevention, College of Public Health, Medical and Veterinary Sciences, James Cook University, Cairns, QLD Australia; 2 Department of Psychology and Sport and Exercise Science, College of Healthcare Sciences, Division of Tropical Health and Medicine, James Cook University, Cairns, QLD Australia; 3 School of Allied Health, Faculty of Health Sciences, Australian Catholic University, Sydney, NSW Australia; 4 Prevention Research Collaboration, Sydney School of Public Health, University of Sydney, Sydney, NSW Australia

**Keywords:** Indigenous, Physical activity, Intervention, Chronic disease, Australia

## Abstract

**Background:**

Aboriginal and Torres Strait Islander people have higher rates of chronic disease and a lower life expectancy than non-Indigenous Australians. In non-urban areas these health disparities are even larger. The aim of this qualitative study was to explore perceived barriers and enablers to attending an eight-week physical activity program in a rural and regional setting which aimed to improve health outcomes, but had a low attendance rate.

**Methods:**

Thirty-four Indigenous Australians participated in the intervention from the rural (*n* = 12) and the regional (*n* = 22) community. Qualitative semi-structured individual interviews were conducted at the follow-up health assessments with 12 participants. A thematic network analysis was undertaken to examine the barriers and enablers to participation in the program.

**Results:**

Overall, there were positive attitudes to, and high levels of motivation towards, the physical activity program. Enablers to participation were the inclusion of family members, no financial cost and a good relationship with the principal investigator, which was strengthened by the community-based participatory approach to the program design. Barriers to program attendance were mostly beyond the control of the individuals, such as ‘sorry business’, needing to travel away from the community and lack of community infrastructure.

**Conclusions:**

More consideration is needed prior to implementation of programs to understand how community-specific barriers and enablers will affect attendance to the program.

**Trial registration:**

ACTRN12616000497404. Registered 18 April 2016.

## Background

Indigenous Australians have a lower life expectancy compared to non-Indigenous Australians, mainly due to higher rates of preventable chronic diseases [1–3]. Chronic diseases, such as type 2 diabetes and cardiovascular disease, are the leading cause of death for Aboriginal and Torres Strait Islander people [1]. Inequalities in health are further exacerbated by the location in which people reside, as rural and remote communities do not have the same opportunities to access services, education and employment [4]. The location and accessibility to Indigenous communities can also prove to be challenging when undertaking research into the health inequalities with Indigenous Australians [5].

There is a limited amount of research conducted with socially disadvantaged groups [6, 7], fundamentally attributed to the continuous struggle for researchers to access, engage with and retain participants [7]. Indigenous Australians are unique in this regard as they are considered to be socially disadvantaged, however, there are concerns of being over-researched without corresponding health improvements [8]. Reasons for poor participation rates in ‘hard-to-reach’ populations can include mistrust in research and fear of exploitation [7], lack of education [9] and generally low population numbers compared to non-Indigenous people [10]. Conducting physical activity research with Indigenous Australians is challenging, but not impossible and the potential benefits could be significant.

A recent systematic review investigated the effects on health outcomes of physical activity programs for Indigenous people in Australia and New Zealand [11]. While there was some evidence to suggest these programs improved health outcomes, most of the 13 studies in the review reported issues with recruitment, retention and adherence to the programs. For instance, several studies had less than 60% of participants complete the program, or failing to have complete data sets [12–16]. A randomised controlled trial by Biddle et al. [17] had an attendance rate of more than 80% to a physical activity intervention which involved small sided (small number of players) games over a four week period. Biddle and colleagues collected written feedback from the participants about their reasons for attending the program in which they stated that they had fun and enjoyed doing the exercise program.

In addition to a quantitative evaluation, one of the studies in the review, a 12-week fitness program for Aboriginal and Torres Strait Islander women [13], also had a qualitative component to identify perceived barriers and facilitators to the attendance of the program [18]. Identified barriers included personal health, access to transport and competing obligations. In regards to logistics and attendance, transportation has also been recognised by Davey et al. [19] as a potential barrier to participation in an Indigenous cardiopulmonary rehabilitation program. The 8-week rehabilitation program involved twice weekly training sessions which were delivered at a gym in a private physiotherapy practice. Free transport to the practice was organised for the participants who would otherwise have had difficulties attending. Additional factors that are known to affect attendance to programs in rural areas are the need to travel away out of the community for work, medical appointments and education, as potential participants would be away for a substantial duration of the week [14].

Understanding the barriers and enablers to participation in physical activity programs can assist researchers in interpreting contextual factors that influence the implementation of and attendance to a program [20]. Thus, the aim of this study was to explore the barriers and enablers to participate in a community-tailored physical activity intervention for Indigenous Australians in a rural and regional setting in Far North Queensland.

## Methods

### Settings and recruitment of participants

The two intervention sites were located in a rural and regional setting in Far North Queensland, Australia. The communities are not identified in this paper to protect the participants’ anonymity. To be included in the study participants had to be Aboriginal or Torres Strait Islanders aged 18–45 who have a chronic disease or a risk factor for chronic disease. Based on the definitions by the Australian Institute of Health and Welfare Guidelines [21], risk factors for chronic diseases include a BMI of or greater than 30, a waist circumference of or higher than 94 cm for men and 80 cm for women and less than 150 min of physical activity per week. Participants were excluded if they were pregnant or advised by a doctor not to engage in physical activity.

**Fig. 1:**
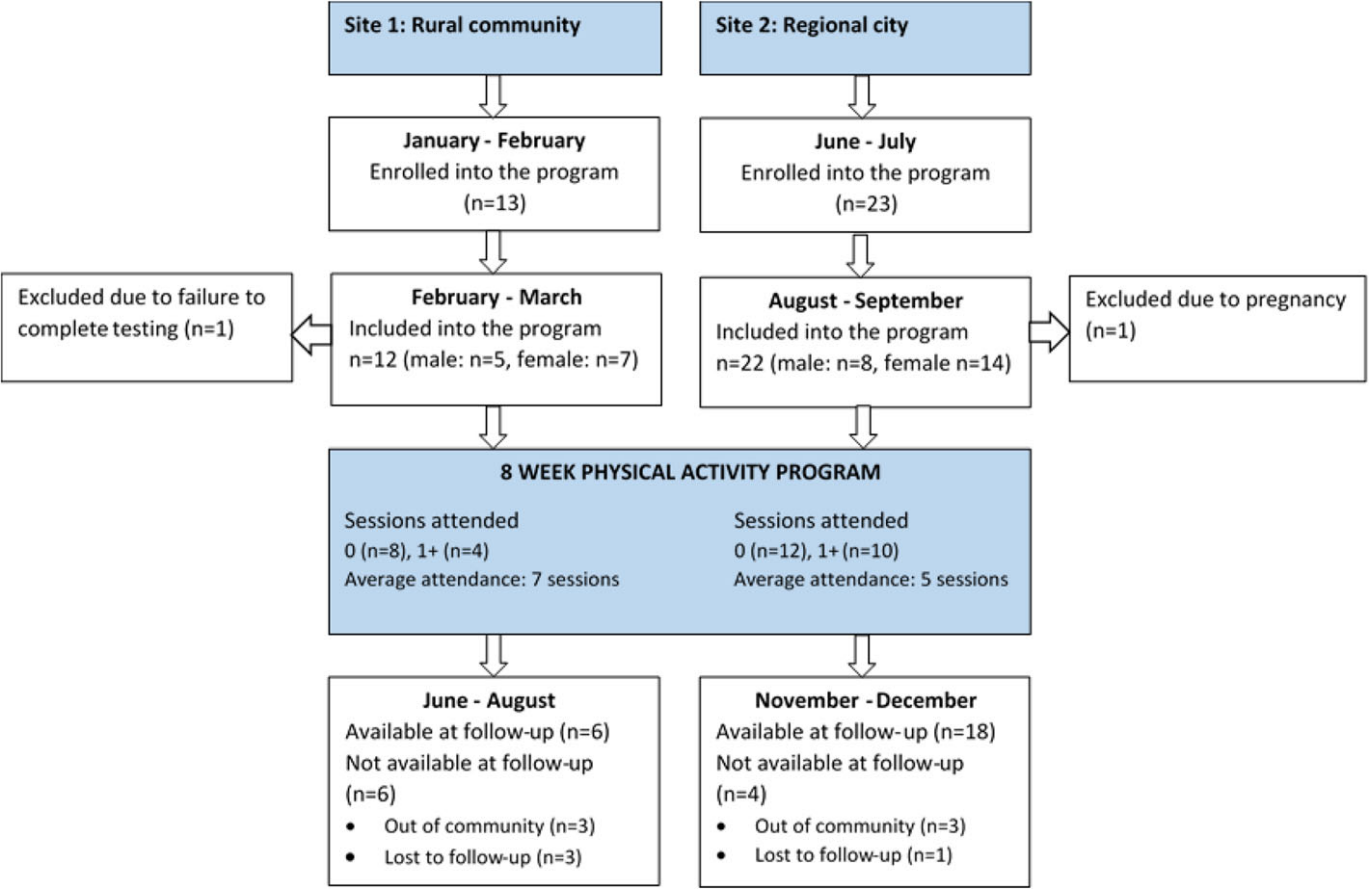
Flowchart of participant recruitment and attendance in each community

The program commenced in January 2016 in the rural community and in July 2016 in the regional location. At the commencement of the study, 34 participants were enrolled. A flowchart of participant recruitment and attendance is provided in Fig. 1.

### Interview guide development

Interviews were conducted at the post-program assessments. The interviews were loosely based on the Health Belief Model [22], as it is one of the most widely recognised conceptual frameworks for health behaviours [22–24]. The Health Belief Model was selected as it was theorised that individuals would be more likely to voluntarily engage in the physical activity program if their current behaviour is perceived as a threat to their health. An interview guide was created to assist in the delivery of semi-structured interviews and included open-ended questions, which were designed to prompt responses relating to experiences with physical activity, the program delivery and future directions for interventions. The interview guide included the following themes:

1. What was your experience with the program?
2. What could have been made better about the program?
3. What would you like to see happen in the future?
4. Is there anything else you would like to comment on?

Due to the complexity of a non-Indigenous researcher working with an Indigenous population group, the interview guide had limited questions to allow a better exploration of previously unidentified themes that may arise.

### Procedure

An 8-week physical activity program was implemented in two communities. The free program was delivered through one-hour face-to-face group sessions, four times per week for eight weeks. Each week there were two sessions with vigorous activities and two with moderate intensity activities, delivered by a qualified sport and exercise scientist (principal investigator). The vigorous intensity sessions included small-sided team sports (small number of players per side), such as soccer and touch football and circuit training with adaptations made to account for individual fitness levels. The remaining two sessions were self-paced moderate intensity walking sessions. After the program, participants were invited to partake in one-on-one interviews, regardless of their attendance to exercise sessions. Interviews were mostly conducted at the participants’ house by the principal investigator (PI) who already had established relationships with the participants.

### Data treatment and analysis

All interviews were digitally recorded by the PI and transcribed professionally by an external transcription service. Transcription files were presented in Microsoft Word and later uploaded into NVivo 11 (QSR International, Australia) for analysis. Interview transcriptions were read by the PI while listening to the corresponding audio file to cross-validate the content and to ensure the context was captured accurately. The interviews were coded and examined independently by a second person (CM) for a thematic analysis. The thematic network analysis was aimed at identifying global, organisational and basic themes [25], which were also compared between the two communities. The analysis involved coding the raw material into specific topics or words, such as ‘money’, ‘time’ and ‘work’ in order to reduce the data. The next step of the analysis involved clustering the codes to identify the organizing themes. The three themes were labelled as ‘barriers’, enablers’, and ‘suggestions for future programs’. Finally, the thematic networks were constructed and arranged for further exploration and interpretation. To compare the findings between the rural and regional communities, the codes within the themes were reviewed and highlighted in two distinct colours to examine the frequency of appearance. The global theme of the data was named as ‘participation in physical activity’ as it best described the overall context of the findings.

## Results

### Participants

Table 1 provides the baseline anthropometric measures for all participants of the intervention. Many participants were unemployed (*n* = 12) and annual gross household income was frequently less than $20,000 (*n* = 7) or less than $60,000 (n = 12). Seven participants had not completed year 12 and 17 participants had completed a TAFE or university degree. Twenty-seven of the 33 respondents (missing n = 1) rated their general health either good (*n* = 16), fair (*n* = 9) or poor (*n* = 2). Only two participants rated their health as excellent. Quantitative results from the program will be provided elsewhere in an additional publication.

**Table 1:** Baseline anthropometric measures of the communities separately, and combined, Queensland, Australia, 2016

		Rural community (*N* = 10)	Regional community (*N* = 22)	Combined (*N* = 32)
		Mean (SD)	Mean (SD)	Mean (SD)
Age		36.6 (6.89)	29.27 (6.0)	31.56 (7.07)
Anthropometrics	Height (cm)	162.5 (9.14)	169.86 (9.10)	167.28 (9.77)
	Weight (kg)	85.81 (12.05)	88.12 (15.61)	87.4 (14.44)
	BMI (kg/m^2^)	32.73 (2.55)	30.53 (4.81)	31.21 (4.32)
	Bodyfat (%)	34.14 (7.99)	34.07 (9.15)	34.09 (8.67)
	6MWT (m)	455 (95.45)	509.72 (65.54)	492.62 (78.86)
	Waist circumference (cm)	104.2 (6.77)	98.59 (11.75)	100.34 (10.67)
	Hip circumference (cm)	105.45 (3.93)	105.15 (10.49)	105.25 (8.89)
Blood pressure	Systolic BP (mmHg)	126.8 (9.30)	124.66 (11.36)^a^	125.35 (10.63)
	Diastolic BP (mmHg)	84.1 (10.22)	81.33 (7.40)^a^	82.82 (8.34)

*6MWT* 6 Minute Walk Test, *BMI* body mass index, *BP* blood pressure, ^a^ Data was not available for one participant

Overall, attendance to the program was low. In the rural community, eight people who enrolled in the program did not attend a single training session, and four attended an average of seven sessions during the eight weeks. In the regional city, twelve participants did not attend a single training session, while the remaining ten participants attended an average of five sessions.

### Motivations to participate in the program

The motivations to join the physical activity program were different between the rural and regional community. During the analysis, the regional community had a sub-theme develop in regards to motivations of health-related outcomes such as weight loss and fitness. For example, reasons to join included ‘To lose weight, get fit and be more active’ (Female 6, Regional) and ‘Weight loss and to get more into physical activity’ (Male 1, Regional). For one participant, weight loss was a means of controlling the progression of her chronic disease: ‘Well, with the current condition I’m under it is very important that I control my weight…I’ve got diabetes’ (Female 5, Regional). The rural community members more explicitly stated motivations to participate were due to chronic diseases. One participant stated ‘I'm determined not to be a [chronic disease] statistic… I'm pretty determined to break the cycle’ (Female 1, Rural), and another explained ‘she [another participant] wanted to do it … her chronic disease wasn’t looking good so she had to start making changes’ (Female 4, Rural).

### Barriers to attendance

Many of the men who enrolled in the program stated that their main reason for non-attendance to activities was work commitments. Two of the participants who worked in the rural community stated ‘I do work away, all the time up the Cape’ (Male 2, Rural) while another often did shift work: ‘Yes, well, at that time I was doing all day shifts at work and the timing was sort of out’ (Male 3, Rural). Both men were highly motivated to participate and attended the follow-up data collection, despite not attending a single training session. The men acknowledged their disappointment that they did not get any benefit from the program. One of the men in the regional community, who was also a university student, found it hard to attend as his work required him to travel away regularly, ‘I think the biggest barriers for me are work, study and travel’ (Male 1, Regional).

There were logistical barriers identified in the interviews, which were unique to the communities. In the regional community, logistical problems were mainly the timing of the class (despite being suggested by the participants), whereas the rural community members experienced problems with access to transport. The rural community also had issues with access to facilities. During the first four weeks of the program, no indoor facilities were available and classes were regularly cancelled due to rain. ‘You’ve got to take into consideration that a lot of outdoor activities can’t be run on a regular basis sometimes, due to - obviously we live in the tropics, the wet tropics. That’s why it’s called wet, as - it can set in for days, months sometimes’ (Female 4, Rural). Despite being able to access a secondary facility five kilometres away, there were barriers in regards to access to transport to attend the classes. ‘But organising stuff, even when there is wet [weather], we don’t have indoor facilities…You’ve got to go in town or you’ve got to access a facility - fitness facilities, and then they cost money. Every time you want to do something, it costs money’ (Female 4, Rural). There was a lack of public transport services and ‘not everybody has access to a car’ (Female 4, Rural).

There were other barriers to program implementation and attendance in the rural community, such as ‘sorry business’, which is a term used by Indigenous Australians that refers to a time of mourning in the community, following the death of a person. The process of mourning can take place over extended periods, and the intensity of the mourning is reflective of the importance of the individual that has passed away, respectively the importance of his or her family. ‘We run a program - we’ve always tried to start it with a start date. Obviously because of the weather, obviously because of sorry business or something, we can never start it when we want to start it. It got put off as much as we could. Then when it got up and running, by the time it was finishing, that’s when they started walking in the door’ (Female 4, Rural). An identified sub-theme in the rural community was menstruation being a barrier to female participation. ‘Sometimes women won’t participate if they’re menstruating. I know quite a few say they can’t’ (Female 4, Rural). Although there was no further elaboration from other participants in the interviews on this particular issue, several personal communications received prior to program sessions indicated that the reason for non-participation was menstruation. Specific concerns reported included feeling uncomfortable exercising, cramping and fatigue and concerns regarding hygiene in the rain.

A lack of family and peer support were identified as a barrier to participating in physical activity. One of the participants who had a young family and a partner that worked away explained: ‘I think the only thing, sometimes I feel depressed there is not enough support from my partner, which is something that I’m wanting more is his support’ (Female 6, Regional). A male participant explained his hardship of needing to take control of his health conditions and the effect it has on his family. ‘I suppose the receptive of the rest of the family is probably your key stumbling block if there’s one … see I’ve got a wife and four kids so it’s like I’ve got to have a separate meal’. He also expressed his feelings toward the impact that health choices have on his children: ‘because I think she’s borderline [his daughter] – at the age of 20, borderline diabetic, and we try to do that with her, but – yeah – sort of heartbreaking when they get emotionally upset because they’re not allowed to go and enjoy what they want to enjoy’ (Male 3, Rural).

The lack of peer support and family also transitioned into themes of shame and stigma around undertaking physical activity. There were previous negative experiences in regards to people wanting to exercise for their health. ‘Now or prior to the last five years, exercise was deemed as people who wanted to look good. There was always this negative attitude. Because I think obesity has become a norm in most – in the cultures now. You either need to look fat, so everyone stays fat, and if you want to lose fat, you're deemed as being sick. If you want to look good for the sake of being healthy, people think that you’re better than anybody else. Whereas I think the culture is shifting a little bit now, or the mentality is shifting’ (Male 1, Regional).

### Enablers to participation

**Fig. 2:**
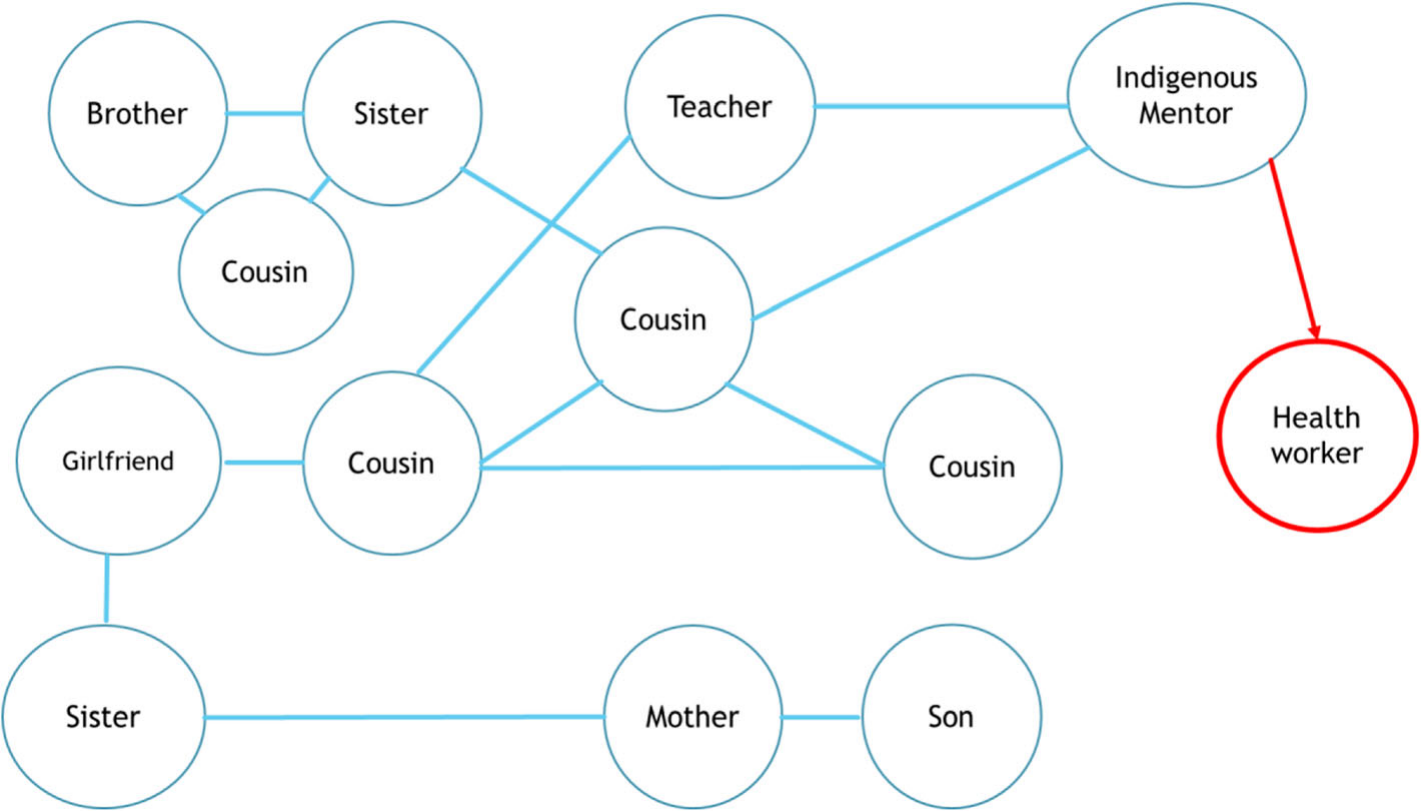
A kinship map of the participants from the rural community. The health worker was not local to the community, but worked with the Indigenous mentor

Several participants mentioned finances and money during the interviews as barriers to participate in physical activity and that having a free program acted as an enabler. Reasons for joining were because ‘it is good that it doesn’t cost money. Everything else costs money’ (Female 7, Regional). Unemployment was as a financial barrier to undertaking exercise as ‘Money is a major barrier for myself. Only because you’re still looking for work’ (Female 3, Regional) and ‘it was free … Yeah, I couldn’t afford it, no money, no job’ (Female 2, Regional). An identified enabler to joining in the physical activity program was the inclusion of family members into the activities. Figure 2 provides a kinship map of the participants in the rural community. Before the program, only the Indigenous mentor was known to the principal investigator. Despite flyers being displayed in the community, recruitment tended to occur via snowballing methods through family members. In the regional community, five members from the same family participated in the program. One of the family members stated ‘It just made it all the better that it was family involved… And yourself of course. You’re family’ (Female 3, Regional).

Aside from family participation, peer support and guidance also assisted with facilitating participation. One person admitted to feeling uncomfortable when exercising on her own due to embarrassment: ‘It's just if I'm by myself in public and I'm going for a jog on the road, I feel very self-conscious … Look at this fat chick she’s jogging, what the hell’ (Female 2, Regional). Another participant stated they needed support and guidance from a qualified person, ‘I tend to do things alone, and I don’t know if I’m doing it right or not. Whereas if you’ve got somebody there who can guide you, you sort of have a better understanding of the process … most of the time I go to the gym, and one of the fears I have is not knowing if I’m actually doing it right. You know that as you’re probably aware, you can hurt yourself…so I tend to do a lot of jogging’ (Male 1, Regional).

Themes of resilience also emerged in regards to the lack of peer support. It was encouraging to hear statements such as: ‘You have, yeah, those kind of people that would think [exercising is a disconnecting activity] like that, but, you know, just got to ignore them and do what’s best for you, I guess … Turn around and tell them where to go, say this is my life, I’m not going to end up with a chronic disease, are you going to look after me when I’m on dialysis or whatever?’ (Female 4, Rural) and ‘I think for me that’s what I’d like to see, is that exercising becomes the norm for our people … my mum comes from the dormitory [stolen generation] [26], so exercising for her was never the norm. I hope I’ve broken the cycle now, my kids will start to see it as more normal’ (Female 1, Rural).

An enabler to participation in the physical activity program was the participants’ relationship with the researcher. Many primary concerns to program implementation in the rural community revolved around the principal investigator being an ‘outsider’, female and non-Indigenous. When participants were asked about whether having a non-Indigenous female run the program could have been an issue, many positive responses were received. One of the male participants explained his thoughts on the principal investigator, focusing on the relationship and level of comfort: ‘Of course, because you really need to have a good relationship and it eliminates the shame factor. Because sometimes you're a bit too embarrassed, even though if you can’t – if you don’t have the strength or the capacity to do what you want to do or achieve’ (Male 1, Regional).

For one of the female participants, having a female principal investigator was seen as an enabler to participation: ‘Not really, when I met you, I was like yeah this is going to be easy. If it was like someone in their 30s, 35 and not like a male, yeah I'm probably not going to rock up to your session if they weren’t so welcoming like you are’ (Female 2, Regional).

Although there was not a negative reaction to the principal investigator being a non-Indigenous female, the need for a male role model was evident, particularly in the rural community. The principal investigator felt comfortable and welcome in the community, however, there were suggestions for future research. For instance, one of the men responded by saying there was a need for male role models: ‘To some extent yeah, a possibility. I mean it doesn’t matter … especially in communities, having a male would benefit, because a lot of the times, when you use girls, the girls always turn up to a lot of your stuff. But the blokes won’t because there’s too many women there. But if there was a bloke there and he sort of did it together, maybe they might turn up’ (Male 2, Rural). In agreement, another male participant also offered his suggestion to increase attendance: ‘Just that it could have been a lot more user friendly in regards to that. I mean, I’m not saying that you’re an uncomfortable person to talk to. You’re very comfortable. But that’s just me. Other people might find it uncomfortable and it’s like role modelling too. So, if you’ve got an – even not so much of a young male or an older male who’s got some problems, health problems, they can actually role model or they’ve come from a background where there’s bad health in the families and so forth’ (Male 3, Rural).

The need for male role models was further emphasised by higher program attendance by women. One woman suggested ‘I think maybe it needs to have a male and a female, because it seemed to have worked more so for the women, than for the men’ (Female 1, Rural) whilst another believed ‘Culture is still focused on the men’s and women’s stuff … we did have a men’s group, but they do a lot of social outings. That’s something we could’ve tapped into’ (Female 4, Rural). It would have been ideal to have a male involved in the program, as this was an issue discussed during the study design, it was extremely difficult to involve a male Indigenous role model. This was due to the lack of male Indigenous health workers and qualified people to run physical activities.

## Discussion

This study sought to explore Aboriginal and Torres Strait Islander participants’ perceived barriers and enablers to attending a community-tailored physical activity program implemented in a rural and a regional setting. There were unique themes identified within the communities in regards to the barriers to attendance, however, the motivations to participate were similar across both sites. Firstly, the rural community had a lack of provisional infrastructure and access to resources. This impacted the program delivery and attendance due to inadequate access to an undercover facility during wet weather events.

### Barriers to participation

There was a contrast between the logistical barriers between the communities where participants in the regional community were unable to attend due to poor timing, as opposed to those in the rural setting for whom lack of access to transport was a major barrier. The rural participants also expressed the importance of having both female and male role models for community members. For many their main motivation to participate in the program was that they saw physical activity as a means for prevention and management of chronic disease. In the regional community motivations were more centred on physical activity-related outcomes of weight loss and increased fitness. Participants from both communities highlighted the importance of support from their family and peers with their journey to becoming more active.

Evaluations of physical activity interventions with Indigenous populations have reported several barriers to participation, which include the transient nature of the population, logistic issues and *shame* [27]. The Indigenous Australian population is more mobile than the remainder of the Australian population, as they often need to leave their homes to access medical services or for cultural obligations such as to attend funerals [28]. Particularly in smaller communities Indigenous people often need to relocate to larger cities to find employment opportunities [14]. For the rural community in this study, the need to travel to a larger city for personal, or family medical reasons was a frequent reason for non-attendance.

Logistical issues, mainly in regards to transportation, are also known barriers to attendance to research programs [18]. Davey et al. [19] recognised access to transportation as an issue and included free transportation to exercise sessions, which was used by the majority of the participants and consequently attributed to retaining participants. In the context of this study, access to free transport was offered at both sites, but car-pooling was more common in the rural community due to the lack of public transportation. In the regional community, the provision of free transport was difficult due to the geographical spread of participants and due to the principal investigator also needing to conduct the exercise sessions. There were some participants who did use public bus services to attend the program.

The concept of *shame* refers to embarrassment in certain situations and is often due to attention or circumstance, rather than the action by oneself [29]. The most prominent example of *shame* occurring was during a walking group in the rural community, where the principal investigator was asked to walk on the opposite side of a participant to block her from the view of men who were sitting across the road. Thompson et al. [30] examined the cultural and social context of physical activity in an urban Aboriginal population in Melbourne. A finding was that the Aboriginal people tended to view physical activity in three different aspects of everyday activities, sports and exercise. Everyday activities are described as necessary and performed for the benefit of the family and community, such as mowing the lawn or playing with children. Sporting competitions which can include individual and team disciplines are highly valued as the player represents the community and is a source of community pride and esteem. However, exercise is viewed as something conducted individually and specifically for personal fitness and health. In this context, the desire to personally improve oneself through exercise can be looked upon as a shameful and disconnecting experience by the community as it is often undertaken separately from family and community. Similar to our findings, Thompson et al. [30] reported that there were some elements of shame around exercising to improve health. The involvement of family in the exercise sessions may have reduced the shame associated with participation and acted as an enabler to participation.

### Enablers to participation

Enablers to participation in the physical activity program primarily revolved around relationships to the other participants and with the principal investigator. In the rural community, all participants had a kinship connection with at least one other member of the program. It became evident that recruitment to the project was largely through kinship connections, rather than through the wider advertisement throughout the community. In the regional community, kinship connections were also an obvious factor in recruitment. One family had five family members involved, while another family had three sisters participate. The flexibility in the program delivery was also well-suited to participants who were the primary carer for children or dependant adults. It was often observed that participants would bring their children to training sessions, and in one instance a participant brought her dependant adult mother to a class. During the walking groups, some women would bring prams or bikes for their children to join in with the activity. There were also several community members who were not part of the program, but who began to attend training with the encouragement of the existing participants.

The fact that the principal investigator who ran the physical activity intervention in the two communities was a non-local, non-Indigenous female was seen as a potential barrier to conducting the research. However, positive feedback was received and participants often ‘vouched’ for the principal investigator during the recruitment of their family members. The only barrier with the principal investigator conducting classes was the lack of mentorship for the men who were involved in the program. This was highlighted during the interviews with the members of the rural community. There was an understanding that it is challenging to find Indigenous men to assist with the program, especially in a voluntary role as it would have been with this project. It is important to be aware of the cultural ideas of men’s and women’s business which refers to certain customs and practices in Indigenous communities that need to be undertaken by men and women separately [31]. Therefore, researchers should aim to have mentors from both genders.

### Strengths

The 8-week physical activity intervention lead to significant improvements in health outcomes, however, attendance was low across both communities. The research project utilised a participatory action research approach [32] and was guided by local Indigenous mentors, in conjunction with a local Aboriginal health organisation. The support from Indigenous mentors was invaluable as they provided a more in-depth understanding of the broader constructs of the community and the cultural ideologies to perform culturally appropriate research. Another strength of the research was the relationship between the principal investigator and the participants. Although the attendance rate was low, the responsiveness of participants was high in terms of availability for follow up assessments and interviews. Originally, there was no qualitative evaluation component planned for the physical activity program. However, after low recruitment and attendance rates were observed during the first few weeks of the program, the qualitative component was added to create a better understanding of the factors that lead to the low participation rates. This allowed for a better interpretation of the study results, as the interviews offered a rich source of data and new information in regards to improving the implementation of physical activity programs in Far North Queensland.

### Limitations

The theoretical model utilised for the qualitative evaluation is a somewhat limiting model for a complex topic. The Health Belief Model was useful to identify barriers and enablers to program participation, but there are suggestions for future research. A limitation of this model is the lack of focus on external factors, such as access to transport, as opposed to individual factors, which can influence health behaviours [33]. A suggestion for future research would be to include the use of an ecological approach [34], as it could have been useful in this context.

## Conclusion

The aim of this qualitative study was to explore perceived barriers and enablers for a rural and regional Indigenous community to participate in an eight-week physical activity program. Overall, there were positive attitudes and high levels of motivation towards the program. Enablers to participation were the inclusion of family members, no financial cost and a good relationship with the principal investigator. Barriers to program attendance were mostly beyond the control of the individuals, such as cultural obligations, the transient nature of communities and lack of community infrastructure. In conclusion, more consideration is needed before program implementation to understand community-specific barriers and enablers to physical activity programs. Furthermore, more work is needed to better understand how to improve participation rates.
